# AMD3100/CXCR4 Inhibitor

**DOI:** 10.3389/fimmu.2015.00276

**Published:** 2015-06-08

**Authors:** Erik De Clercq

**Affiliations:** ^1^Department of Microbiology and Immunology, Rega Institute for Medical Research, KU Leuven, Leuven, Belgium

**Keywords:** anti-HIV, CXCR4, bicyclam, AMD3100, stem cell

The original bicyclam, JM1657 (JM standing for Johnson Matthey) was discovered as a contaminant in a commercial preparation of monocyclams when evaluated for their anti-HIV activity. The original compound, in which the cyclam rings were tethered by a C–C linkage could not be re-synthesized but launched the synthesis of new bicyclams in which the cyclam moieties were linked through an aliphatic bridge: one of these derivatives, i.e., JM2763, exhibited an anti-HIV activity similar to that of JM1657 (1). The compound was postulated to interfere with the uncoating of HIV, a stage in the replicative cycle of HIV, which was (and still is) ill-defined. A quantum jump in anti-HIV potency was achieved with the synthesis of AMD3100 (AMD standing for AnorMeD) (which was originally called JM3100), where the two cyclam rings are tethered by an aromatic bridge (Figure 1A) (2). The compound was active against HIV in the low nanomolar concentration range and generated considerable commercial interest, although its precise mechanism of action remained enigmatic (3, 4). Finally, the viral glycoprotein gp120 was identified as the molecular target of AMD3100 (5). It appeared to be an indirect target. The direct target was CXCR4, with which gp120 has to interact for HIV to enter the cells. AMD3100 was shown to specifically antagonize CXCR4, and thus to block the entry of the T-lymphotropic HIV strains (6–8). AMD3100 appears to be a highly specific inhibitor of CXCR4 (9): it only blocks, as measured by the Ca^++^ flux, the signal pathway from CXCR4 (Figure 1B) and not that of any other receptor for either CXC- or C–C-chemokines (9). Certain aspartic acid residues play an essential role in the interaction of CXCR4 with AMD3100 (Figure 1C) (10, 11).

**Figure 1:**
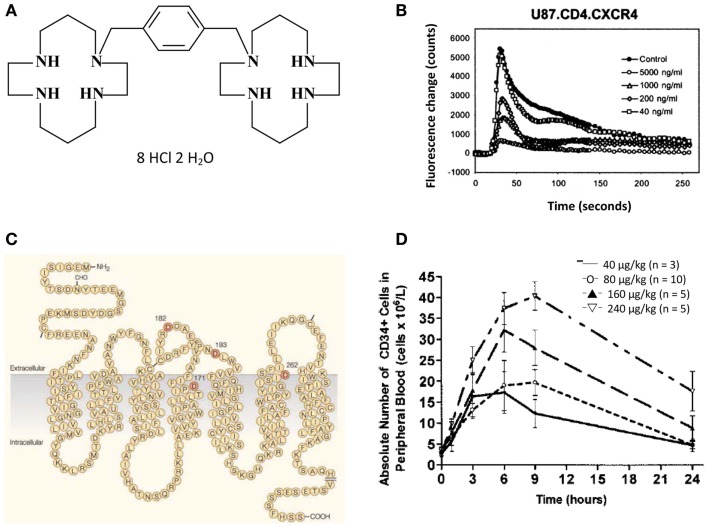
**(A)** Structure of AMD3100. **(B)** Inhibitory effect of AMD3100 on Ca^++^ flux in CXCR4 transfected cells (9). **(C)** The CXCR4 receptor. Crucial aspartic acid residues at positions 171, 182, 193, and 262 in the interaction of CXCR4 with AMD3100 are indicated (11). **(D)** Mobilization of CD34^+^ hematopoietic stem cells (HSCs) by AMD3100 (12).

Within the scope of the potential clinical use of AMD3100 for the treatment of HIV infections, initial phase 1 clinical trials were initiated (13). These studies revealed an increase in the white blood cell (WBC) counts peaking at about 8–10 h after (subcutaneous) injection. These WBCs contained hematopoietic stem cells (HSCs) carrying the CD34 marker (12) (Figure 1D). In fact, the first proof-of-principle that AMD3100 could mobilize hematopoietic stem and progenitor cells was provided by Broxmeyer et al. (14). Thus, the concept was born that AMD3100 (now also called plerixafor or Mozobil^®^) could function as a mobilizer of HSCs. This mobilization is clearly based on the interaction of AMD3100 with CXCR4. CXCR4 is normally the receptor for the chemokine SDF-1 (now called CXCL12), which is responsible for the “homing” of the HSCs in the bone marrow. Under the influence of AMD3100, the HSCs leave the bone marrow to enter the bloodstream where they can be collected and subsequently used for autologous transplantation. In December 2008, Mozobil^®^ was approved by the FDA for this indication in patients with non-Hodgkin’s lymphoma or multiple myeloma. It is used in combination with granulocyte-colony stimulating factor (G-CSF) [for review, see Keating (15)]. For prescribing information, see Ref. (16).

AMD3100 was not further developed for the treatment of HIV infections essentially because of two reasons: (i) AMD3100 was not effective against the M-tropic CCR5 HIV strains, a problem that could be circumvented by the concomitant (oral) use of a CCR5 antagonist, maraviroc (Selzentry^®^), and (ii) it had to be injected subcutaneously, as it was not orally bioavailable. Subcutaneous injection is indeed a problem for long-term administration, and Fuzeon^®^ (enfuvirtide) is the only anti-HIV drug out of more than 25, which has to be administered by injection, and, therefore, not widely used. Attempts to increase the spectrum of AMD3100 derivatives toward M-tropic HIV strains and, particularly, to increase their oral bioavailability led to the synthesis of AMD3465 (17), AMD11070 (18), and various other compounds (19–21), which, however, were not further developed as clinical candidates for treatment of HIV infections. Related CXCR4 antagonists such as KRH-1636 (22), KRH-3955 (23), and T140 analogs (24) were described by Naoki Yamamoto and his colleagues in Japan.

## Conflict of Interest Statement

The author declares that the research was conducted in the absence of any commercial or financial relationships that could be construed as a potential conflict of interest.

## References

[1] De Clercq E Yamamoto N Pauwels R Baba M Schols D Nakashima H Potent and selective inhibition of human immunodeficiency virus (HIV)-1 and HIV-2 replication by a class of bicyclams interacting with a viral uncoating event. Proc Natl Acad Sci U S A (1992) 89:5286–90. 10.1073/pnas.89.12.5286 1608936 PMC49276

[2] De Clercq E Yamamoto N Pauwels R Balzarini J Witvrouw M De Vreese K Highly potent and selective inhibition of human immunodeficiency virus by the bicyclam derivative JM3100. Antimicrob Agents Chemother (1994) 38:668–74. 10.1128/AAC.38.4.668 7913308 PMC284523

[3] De Vreese K Reymen D Griffin P Steinkasserer A Werner G Bridger GJ The bicyclams, a new class of potent human immunodeficiency virus inhibitors, block viral entry after binding. Antiviral Res (1996) 29:209–19. 10.1016/0166-3542(95)00837-3 8739600

[4] Esté JA De Vreese K Witvrouw M Schmit JC Vandamme AM Anné J Antiviral activity of the bicyclam derivative JM3100 against drug-resistant strains of human immunodeficiency virus type 1. Antiviral Res (1996) 29:297–307. 10.1016/0166-3542(95)00936-1 8739608

[5] De Vreese K Kofler-Mongold V Leutgeb C Weber V Vermeire K Schacht S The molecular target of bicyclams, potent inhibitors of human immunodeficiency virus replication. J Virol (1996) 70:689–96. 8551604 10.1128/jvi.70.2.689-696.1996 PMC189868

[6] Schols D Struyf S Van Damme J Esté JA Henson G De Clercq E. Inhibition of T-tropic HIV strains by selective antagonization of the chemokine receptor CXCR4. J Exp Med (1997) 186:1383–8. 10.1084/jem.186.8.1383 9334378 PMC2199084

[7] Schols D Esté JA Henson G De Clercq E. Bicyclams, a class of potent anti-HIV agents, are targeted at the HIV coreceptor fusin/CXCR-4. Antiviral Res (1997) 35:147–56. 10.1016/S0166-3542(97)00025-9 9298754

[8] Donzella GA Schols D Lin SW Esté JA Nagashima KA Maddon PJ AMD3100, a small-molecule inhibitor of HIV-1 entry via the CXCR4 co-receptor. Nat Med (1998) 4:72–7. 10.1038/nm0198-072 9427609

[9] Hatse S Princen K Bridger G De Clercq E Schols D. Chemokine receptor inhibition by AMD3100 is strictly confined to CXCR4. FEBS Lett (2002) 527:255–62. 10.1016/S0014-5793(02)03143-5 12220670

[10] Gerlach LO Skerlj RT Bridger GJ Schwartz TW. Molecular interactions of cyclam and bicyclam non-peptide antagonists with the CXCR4 chemokine receptor. J Biol Chem (2001) 276:14153–60. 10.1074/jbc.M010429200 11154697

[11] Hatse S Princen K Gerlach LO Bridger G Henson G De Clercq E Mutation of Asp(171) and Asp(262) of the chemokine receptor CXCR4 impairs its coreceptor function for human immunodeficiency virus-1 entry and abrogates the antagonistic activity of AMD3100. Mol Pharmacol (2001) 60:164–73. 10.1124/mol.60.1.164 11408611

[12] Liles WC Broxmeyer HE Rodger E Wood B Hübel K Cooper S Mobilization of hematopoietic progenitor cells in healthy volunteers by AMD3100, a CXCR4 antagonist. Blood (2003) 102:2728–30. 10.1182/blood-2003-02-0663 12855591

[13] Hendrix CW Flexner C MacFarland RT Giandomenico C Fuchs EJ Redpath E Pharmacokinetics and safety of AMD-3100, a novel antagonist of the CXCR-4 chemokine receptor, in human volunteers. Antimicrob Agents Chemother (2000) 44:1667–73. 10.1128/AAC.44.6.1667-1673.2000 10817726 PMC89930

[14] Broxmeyer HE Orschell CM Clapp DW Hangoc G Cooper S Plett PA Rapid mobilization of murine and human hematopoietic stem and progenitor cells with AMD3100, a CXCR4 antagonist. J Exp Med (2005) 201:1307–18. 10.1084/jem.20041385 15837815 PMC2213145

[15] Keating GM. Plerixafor: a review of its use in stem-cell mobilization in patients with lymphoma or multiple myeloma. Drugs (2011) 71:1623–47. 10.2165/11206040-000000000-00000 21861545

[16] Available from: http://products.sanofi.us/Mozobil/mozobil.html

[17] Bridger GJ Skerlj RT Hernandez-Abad PE Bogucki DE Wang Z Zhou Y Synthesis and structure-activity relationships of azamacrocyclic C-X-C chemokine receptor 4 antagonists: analogues containing a single azamacrocyclic ring are potent inhibitors of T-cell tropic (X4) HIV-1 replication. J Med Chem (2010) 53:1250–60. 10.1021/jm901530b 20043638

[18] Skerlj RT Bridger GJ Kaller A McEachern EJ Crawford JB Zhou Y Discovery of novel small molecule orally bioavailable C-X-C chemokine receptor 4 antagonists that are potent inhibitors of T-tropic (X4) HIV-1 replication. J Med Chem (2010) 53:3376–88. 10.1021/jm100073m 20297846

[19] Skerlj R Bridger G McEachern E Harwig C Smith C Wilson T Synthesis and SAR of novel CXCR4 antagonists that are potent inhibitors of T tropic (X4) HIV-1 replication. Bioorg Med Chem Lett (2011) 21:262–6. 10.1016/j.bmcl.2010.11.023 21109432

[20] Skerlj R Bridger G McEachern E Harwig C Smith C Kaller A Design of novel CXCR4 antagonists that are potent inhibitors of T-tropic (X4) HIV-1 replication. Bioorg Med Chem Lett (2011) 21:1313–8. 10.1016/j.bmcl.2011.01.021 21295470

[21] Khan A Nicholson G Greenman J Madden L McRobbie G Pannecouque C Binding optimization through coordination chemistry: CXCR4 chemokine receptor antagonists from ultrarigid metal complexes. J Am Chem Soc (2009) 131:3416–7. 10.1021/ja807921k 19231846 PMC2680178

[22] Ichiyama K Yokoyama-Kumakura S Tanaka Y Tanaka R Hirose K Bannai K A duodenally absorbable CXC chemokine receptor 4 antagonist, KRH-1636, exhibits a potent and selective anti-HIV-1 activity. Proc Natl Acad Sci U S A (2003) 100:4185–90. 10.1073/pnas.0630420100 12642669 PMC153550

[23] Murakami T Kumakura S Yamazaki T Tanaka R Hamatake M Okuma K The novel CXCR4 antagonist KRH-3955 is an orally bioavailable and extremely potent inhibitor of human immunodeficiency virus type 1 infection: comparative studies with AMD3100. Antimicrob Agents Chemother (2009) 53:2940–8. 10.1128/AAC.01727-08 19451305 PMC2704660

[24] Tamamura H Tsutsumi H Masuno H Mizokami S Hiramatsu K Wang Z Development of a linear type of low molecular weight CXCR4 antagonists based on T140 analogs. Org Biomol Chem (2006) 4:2354–7. 10.1039/b603818b 16763678

